# Fruit and Vegetable Consumption Interacts With *HNF1A* Variants on the C-Reactive Protein

**DOI:** 10.3389/fnut.2022.900867

**Published:** 2022-07-07

**Authors:** Dayeon Shin, Kyung Won Lee

**Affiliations:** ^1^Department of Food and Nutrition, Inha University, Incheon, South Korea; ^2^Department of Home Economics Education, Korea National University of Education, Cheongju-si, South Korea

**Keywords:** *HNF1A* gene variants, fruit intake, vegetable intake, C-reactive protein, inflammation

## Abstract

Epidemiological studies have demonstrated the inverse association between the intake of fruits and vegetables and inflammation. However, the mechanisms by which inflammation-related genes interact with fruit and vegetable intake and the role of these combinations in inflammation remain unclear. Therefore, we assessed the effect of interactions between fruit and vegetable intake and the hepatic nuclear factor 1 alpha (*HNF1A*) genetic variants on the C-reactive protein (CRP) levels. Baseline data from the Ansan and Ansung Cohort Study of the Korean Genome and Epidemiology Study (KoGES) were used. A total of 7,634 participants (3,700 men and 3,934 women) were included in the analyses. Fruit and vegetable intake was assessed using semi-quantitative food frequency questionnaire data. Genotyping information for *HNF1A* was extracted from the Affymetrix Genome-Wide Human SNP array 5.0. Inflammation was determined after overnight fasting by measuring CRP levels using automated analyzers. Multivariable logistic regression was used to estimate the adjusted odds ratio (AOR) with a 95% confidence interval (CI). In the fully adjusted model, men and women with the GG genotype of HNF1A rs2393791 and high fruit intake had lower odds of elevated CRP levels compared to those with the AA genotype and low fruit intake (AOR 0.50, 95% CI 0.38–0.67; AOR 0.73, 95% CI 0.55–0.97, respectively). Men and women with the rs2393791 GG genotype and high vegetable intake had lower odds of having elevated CRP levels compared to those with the AA genotype and low fruit intake (AOR 0.57, 95% CI 0.43–0.75; AOR 0.65, 95% CI 0.49–0.86, respectively). Men and women with the GG genotype and high total fruit and vegetable intake had lower odds of having elevated CRP levels. These findings indicate that fruit and vegetable intake interacts with *HNF1A* genetic polymorphisms, consequently influencing the inflammation levels.

## Introduction

A growing body of evidence indicates that inflammation plays a crucial role in the pathogenesis of several diseases, such as cardiovascular diseases (1), metabolic diseases (2), type 2 diabetes (3), and cancer (4). Inflammation is closely associated with dietary factors (5–7). Specifically, antioxidants, such as β-carotene, vitamin C, fiber, and other phytochemicals abundant in fruits and vegetables, have been inversely associated with inflammation markers, such as C-reactive protein (CRP) (8–10). In Korea, the current recommended intake of fruits and vegetables is more than 500 grams per day (11), underscoring the beneficial effects of consuming fruits and vegetables to optimize nutritional intake and offer various health benefits, such as reducing disease risk and maximizing positive health outcomes (12). Fruit and vegetable intake reportedly reduced inflammation in healthy, well-nourished, non-smoking German men in a randomized controlled trial, which indicated that those who consumed eight servings of fruits and vegetables a day had significantly lower plasma CRP levels within 4 weeks compared to those who only consumed two servings a day (13).

Strong evidence has indicated that genetic factors play a vital role in the onset of inflammation. Hepatic nuclear factor 1 alpha (*HNF1A*) encodes the transcription factor HNF-1 α, an important transcriptional regulator of various hepatic genes (14). HNF-1α binding sites exist in the CRP promoter region (15), and this illustrates the role of *HNF1A* expression in regulating CRP expression (16). Genetic polymorphisms in the *HNF1A* gene are closely associated with CRP. Carriers of rs2259816 A allele in *HNF1A* have been associated with approximately 15% decreased CRP levels in the German population (17). In healthy Filipino women, carriers of the rs7305618 T allele in *HNF1A* were positively associated with plasma CRP levels (β = 0.288, *P* = 1.0 ×10^−8^) (18).

Although the relationship between fruit and vegetable intake and inflammation has been investigated (13), the mechanisms by which inflammation-related genes interact with fruit and vegetable intake and the role of these combinations in inflammation remain unclear. Therefore, gene–diet interactions must be investigated to understand the underlying pathophysiology of the disease, which could potentially be useful in inflammation-based assessment for the risk stratification. Therefore, we assessed the effects of fruit and vegetable intake and *HNF1A* genetic variants on inflammation.

## Methods

### Data Set

#### Study Design and Participants

The data set from the Ansan-Ansung Cohort Study of the Korean Genome and Epidemiology Study (KoGES), an ongoing large-scale prospective study conducted by the Korea National Institute of Health, was used for this study. The Ansan-Ansung study began in 2001–2002 (baseline) to investigate genetic and environmental risk factors, including dietary factors, that affect chronic diseases in the Korean population (19). Initially, 10,030 adults (40–69 years old) residing in Ansan (urban) and Ansung (rural) cities were included in the study. The participants were followed up biannually, and their follow-up data collected up until 2012 was included.

From the 10,030 participants at baseline examination, we excluded those without genotyping data (*n* = 1,633), who had a diagnosis of myocardial infarction, coronary artery or congestive heart disease, stroke, or cancer (*n* = 350), no dietary information or energy intake <500 kcal/day or >5,000 kcal/day (*n* = 342), and no information on BMI or lifestyle variables (*n* = 71). The final analytical dataset comprised 7,634 individuals (3,700 men and 3,934 women). The protocol was reviewed and approved by the Institutional Review Board (IRB) of Inha University, Korea, on February 18, 2022 (IRB no. 220215-1A).

#### Dietary Assessment

Dietary data were collected using a 103-item semi-quantitative food frequency questionnaire (FFQ) by well-trained interviewers during the baseline examination. To assess typical dietary intake, the validated FFQ was developed and utilized for Korean adults in the KoGES. Fruit intake included the consumption of the 12 fruit items: persimmon/dried persimmon, tangerine, melon/oriental melon, banana, pear, apple/apple juice, orange/orange juice, watermelon, peach/prune, strawberry, grape/grape juice, and tomato/tomato juice. Vegetable intake comprised 23 items: Korean cabbage, radish/salted radish, watery kimchi made of sliced radishes/radish water kimchi, other kimchi (such as green onion, leaf mustard, and Korean lettuce kimchi), green pepper, hot pepper leaves, spinach, lettuce, perilla leaves, chives/water parsley, other green vegetables (such as horseradish, crown daisy, mallow, aster, and turnip green), radish (soup or braised), pickled radish, bellflower/deodeok, onion, cabbage/cabbage soup, cucumber, bean sprouts or mungbean sprouts, carrot, carrot juice, pumpkin (soup or juice), zucchini, vegetable juice, bracken/sweet potato leaves, and pickled vegetables (20). Nine possible responses regarding the intake were provided, ranging from never or seldom to ≥ 3 times per day. For the portion size, three options for each food (0.5 serving, 1 serving (standard), and ≥2 servings) were provided. One serving of fruit or vegetable corresponded to 100 g or 70 g, respectively. To calculate the usual intake of fruits and vegetables, the consumption frequency for each fruit and vegetable food item was multiplied by the nutrient content of its corresponding food item based on a nutrient database (CAN-Pro 2.0) developed by the Korean Nutrition Society (21). The separate intake of fruits and vegetables and the total intake of fruits and vegetables were considered for this study.

### Genotyping

**Figure 1:**
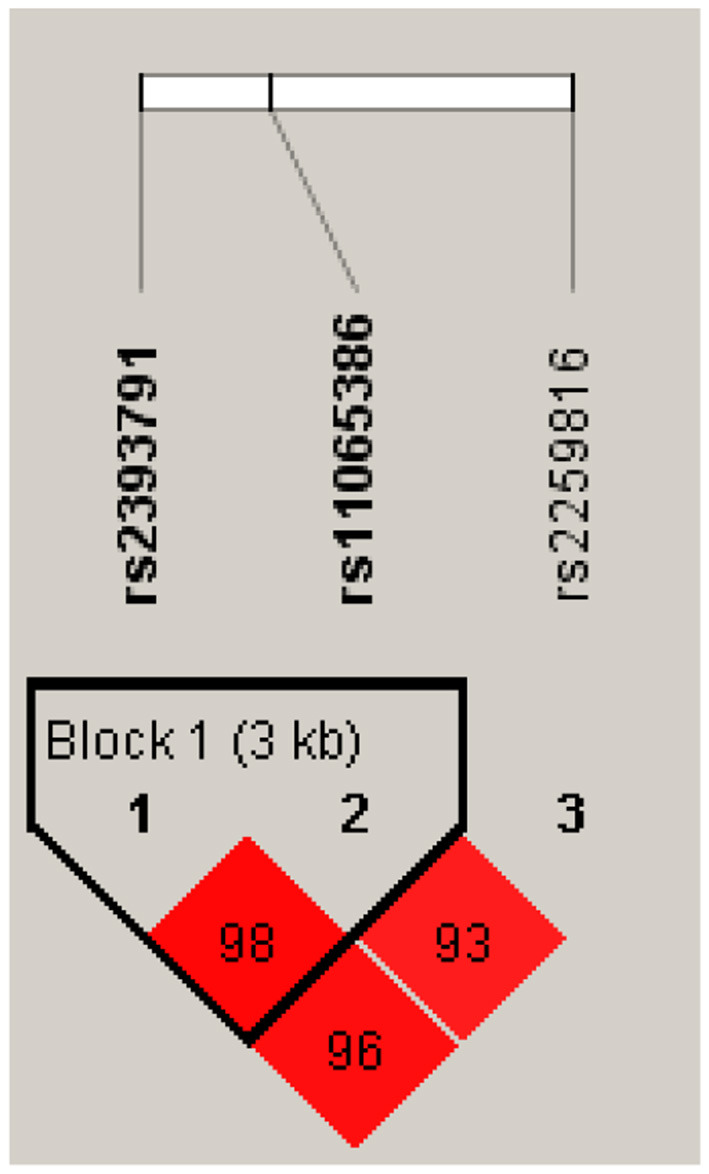
Linkage disequilibrium block of *HNF1A*.

Genomic DNA samples were isolated from whole blood and genotyped using an Affymetrix Genome-Wide Human single nucleotide polymorphism (SNP) array 5.0 (Affymetrix, Inc., Santa Clara, CA, USA). A total of 10,004 KARE study samples were genotyped using this platform. Single nucleotide polymorphisms (SNPs) are the most common type of genetic variations between alleles (22). The quality control procedures were as follows: samples were excluded if they had low genotyping calls (<96%), high heterozygosity, sex inconsistencies, cryptic relatedness, or serious concomitant illness. Markers with high missing gene call rates (>5%), low minor allele frequency (<0.01), or significant deviation from the Hardy–Weinberg equilibrium (P <1 ×10^−6^) were excluded (23). Imputation analysis was performed using IMPUTE program for the Asian HapMap (JPT + CHB) population (release 22/NCBI, build 36, and dbSNP build 126) as a reference panel (23). Three SNPs (rs2393791, rs11065386, and rs2259816) in *HNF1A* were identified, and rs2393791 was chosen for the final analysis based on the results of the tag SNP section (Figure 1).

### Measurement of Inflammation

After overnight fasting, CRP levels were measured using an automated analyzer (CRP: Hitachi Automatic Analyzer 7600, Hitachi, Nittobo, Japan). CRP levels >0.2 mg/dL were considered as elevated (24).

### Statistical Analyses

Descriptive statistics were used to calculate the mean, standard deviations, and frequencies based on CRP status. To assess the relationship between CRP status and demographics, alcohol intake, metabolic equivalent of task (MET) (hours/week), BMI, and fruit and vegetable intake were measured. MET was defined as the amount of oxygen consumed in the resting state and was equal to 3.5 mL O_2_ per kg body weight × min (25).

Multivariable linear regression analyses were performed to determine the association between rs237391 genotype, fruit intake, and vegetable intake with log-transformed CRP after controlling for covariates. Multivariable logistic regression analyses were performed to assess the association of intake of fruits and vegetables and rs2393791 genotypes (AA, GA, GG) with elevated CRP (>0.2 mg/dL) after controlling for covariates. Men and women were divided into groups based on their median intake of fruit (131.3 g and 175.6 g, respectively), vegetable (78 g and 78.4 g, respectively), or total fruit and vegetable (224.1 g and 271.5 g, respectively).

## Results

### General Characteristics of Study Participants

Table 1 presents the descriptive statistics for demographic characteristics, lifestyle factors, rs2393791 genotype, alcohol intake, MET (hours/week), BMI (kg/m^2^), and fruit and vegetable intake. Sex, *HNF1A* rs2393791 genotype, region, and smoking status contributed to significant differences in the CRP status (P <0.05). Participants with CRP >0.2 mg/dL were significantly older, had significantly higher BMI, and had higher total and HDL cholesterol levels than those with CRP ≤ 0.2 mg/dL.

**Table 1:** Characteristics of study participants in accordance with their C-reactive protein levels.

	**C-reactive protein**		**C-reactive protein**		
	**≤0.2 mg/dL (*****n*** **=** **5,204)**		**>** **0.2 mg/dL (*****n*** **=** **2,430)**		
	**n**	**%**	**n**	**%**	***P* value**
Sex		
Men	2,472	(47.5)	1,228	(50.5)	0.0135
Women	2,732	(52.5)	1,202	(49.5)	
rs2393791 genotype		
AA	1,260	(24.2)	760	(31.3)	<0.0001
GA	2,664	(51.2)	1,179	(48.5)	
GG	1,280	(24.6)	491	(20.2)	
Area of residence		
Ansung	2,323	(44.6)	1,163	(47.9)	0.0085
Ansan	2,881	(55.4)	1,267	(52.1)	
Smoking status					
None	3,123	(60.0)	1,340	(55.1)	0.0003
Past	787	(15.1)	405	(16.7)	
Current	1,294	(24.9)	685	(28.2)	
	Mean	SD	Mean	SD	
Age (years)	51.3	(8.7)	53.5	(9)	<0.0001
Alcohol intake (g/day)	9.3	(20.9)	10.3	(24)	0.0693
MET (hours/week)	163.2	(102.9)	161.7	(103.7)	0.5713
BMI (kg/m^2^)	24.2	(3)	25.3	(3.3)	<0.0001
Fruit intake (g/day)	246.4	(283.4)	247.6	(298.3)	0.8681
Vegetable intake (g/day)	101	(90.9)	99	(86.6)	0.3563
Total cholesterol (mg/dL)	196.6	(36.0)	203.5	(39.2)	<0.0001
HDL cholesterol (mg/dL)	50.1	(11.8)	47.7	(11.6)	<0.0001

*P values were based on the chi-square or t-tests for categorical and continuous variables, respectively*.

*SD, standard deviation*.

Demographic characteristics, lifestyle factors, fruit and vegetable intake, and CRP levels by rs2393791 genotype are presented in Table 2. CRP levels significantly differed according to the rs2393791 genotype (*P* <0.0001). CRP was highest in rs2393791 AA genotype (0.30 ± 0.84 mg/dL) compared to that in GA or GG genotypes (vs. 0.22 ± 0.40 vs. 0.19 ± 0.35 mg/dL). Sex, region, smoking status, age, alcohol intake, MET, BMI, and fruit and vegetable intake did not significantly differ according to the rs2393791 genotype.

**Table 2:** Characteristics of study participants by *HNF1A* rs2393791 genotype.

	**rs2393791 Genotype**						
	**AA (*****n*** **=** **2,020)**		**GA (*****n*** **=** **3,843)**		**GG (*****n*** **=** **1,771)**		***P* value**
	**n**	**%**	**n**	**%**	**n**	**%**	
Sex							
Men	1,008	(49.9)	1,829	(47.6)	863	(48.7)	0.2361
Women	1,012	(50.1)	2,014	(52.4)	908	(51.3)	
Region							
Ansung	887	(43.9)	1,800	(46.8)	799	(45.1)	0.0884
Ansan	1,133	(56.1)	2,043	(53.2)	972	(54.9)	
Smoking status							
None	1,174	(58.1)	2,247	(58.5)	1,042	(58.8)	0.9667
Past	311	(15.4)	603	(15.7)	278	(15.7)	
Current	535	(26.5)	993	(25.8)	451	(25.5)	
	Mean	SD	Mean	SD	Mean	SD	*P* value
Age (years)	52	(8.9)	52	(8.8)	51.9	(8.8)	0.9476
Alcohol intake (g/day)	9.5	(20.9)	9.5	(22.5)	9.9	(22)	0.7681
MET (hours/week)	162	(102.7)	162.7	(103.6)	163.5	(102.7)	0.9034
BMI (kg/m^2^)	24.7	(3.2)	24.5	(3.1)	24.7	(3.2)	0.1155
Fruit intake (g/day)	249.2	(299.3)	246.8	(284.2)	243.8	(284)	0.8464
Vegetable intake (g/day)	100.2	(86.6)	100.5	(91.5)	100.3	(88.8)	0.991
C-reactive protein (mg/dL)	0.30	(0.84)	0.22	(0.40)	0.19	(0.35)	<0.0001
Total cholesterol (mg/dL)	197.9	(37.7)	199.2	(36.8)	199.0	(37.4)	0.4639
HDL-cholesterol (mg/dL)	48.9	(11.7)	49.5	(11.9)	49.6	(11.8)	0.1723

*P-value based on the chi-square test or generalized linear model for categorical and continuous variables, respectively*.

### Association of Fruit and Vegetable Intake and rs2393791 With CRP

The results on the association between fruit and vegetable intake and rs2393791 genotypes with log-transformed CRP levels are listed in Table 3. Dietary intake of fruits and vegetables was negatively and significantly associated with log-transformed CRP (β = −0.0001 for fruits, β = −0.0005 for vegetables, and β = −0.126 for rs2393791 genotypes).

**Table 3:** General linear model examining the association between fruit and vegetable intake and *HNF1A* rs2393791 with log-transformed C-reactive protein.

	**Log-transformed C-reactive protein**			
	**β**	**Standard error**	**t value**	***P* value**
Fruit intake (g/day)	−0.0001	0.00005	−2.53	0.0115
Vegetable intake (g/day)	−0.0005	0.00016	−3.41	0.0006
rs2393791 genotypes (AA, GA, GG)	−0.126	0.020	−6.31	<0.0001

### Association of Fruit Intake and rs2393791 Genotype With CRP

Associations of rs2393791 genotype and fruit intake divided by the median intake with elevated CRP (>0.2 mg/dL) by sex are presented in Table 4. Compared to men with the AA genotype of rs2393731 who consumed low fruit, those with the GA or GG genotype had significantly lower odds of elevated inflammation regardless of the fruit intake levels after controlling for covariates such as age, region, smoking, MET, alcohol intake, and BMI. Notably, men with the GG genotype and high fruit intake had the lowest odds of having elevated CRP levels after controlling for covariates (adjusted odds ratios (AOR) 0.50, 95% confidence interval [CI] 0.38–0.67) compared to those with the AA genotype and low fruit intake. In women, compared to those with the rs2393731 AA genotype and low fruit intake, those with the GG genotype and high fruit intake had lower odds of having elevated CRP levels after controlling for covariates (AOR 0.73, 95% CI 0.55–0.97).

**Table 4:** Association of *HNF1A* rs23939791 genotype and fruit intake with inflammation.

**rs2393791 genotype**	**Men (*****n*** **=** **3,700)**			**Women (*****n*** **=** **3,934)**		
	**Fruit intake**	**AOR (95% CI)**	***P* value**	**Fruit intake**	**AOR (95% CI)**	***P* value**
AA	≤ 131.3 g	1.00 (Ref.)		≤ 175.6 g	1.00 (Ref.)	
	>131.3 g	0.81 (0.63–1.05)	0.1088	>175.6 g	1.13 (0.87–1.48)	0.3657
GA	≤ 131.3 g	0.68 (0.54–0.86)	0.0012	≤ 175.6 g	0.73 (0.57–0.92)	0.0086
	>131.3 g	0.63 (0.50–0.79)	0.0001	>175.6 g	0.87 (0.68–1.10)	0.2333
GG	≤ 131.3 g	0.53 (0.40–0.70)	<0.0001	≤ 175.6 g	0.71 (0.53–0.95)	0.0218
	>131.3 g	0.50 (0.38–0.67)	<0.0001	>175.6 g	0.73 (0.55–0.97)	0.0311

*Adjusted for age (years), area of residence (Ansan, Ansung), smoking (none, past, current), MET (hours/week), alcohol intake (g/day), and BMI (kg/m^2^)*.

*AOR, adjusted odds ratio; 95% CI, 95% confidence intervals*.

*Fruit intake was divided by median intake of 131.3 g for men and 175.6 g for women*.

### Association of Vegetable Intake and rs2393791 Genotype With CRP

Associations of rs2393791 genotype and vegetable intake divided by to the median intake with elevated CRP (>0.2 mg/dL) by sex are presented in Table 5. Compared to men with the rs2393731 AA genotype who consumed low amounts of vegetables, those with the GA or GG genotype had significantly lower odds of elevated inflammation regardless of vegetable intake levels after controlling for covariates. Specifically, men with the rs2393791 GG genotype and high vegetable intake had lower odds of having elevated CRP (AOR 0.57, 95% CI 0.43–0.75) after controlling for covariates. In addition, compared to women with the rs2393791 AA genotype and low vegetable intake, those with the GA or GG genotype had lower odds of having elevated CRP, regardless of vegetable intake in the fully adjusted model. Notably, women with the GG genotype and high vegetable intake had the lowest odds of having elevated CRP (AOR 0.65, 95% CI 0.49–0.86) in the fully adjusted model.

**Table 5:** Association of *HNF1A* rs2393791 genotype and vegetable intake with inflammation.

**rs2393791 genotype**	**Men (*****n*** **=** **3,700)**			**Women (*****n*** **=** **3,934)**		
	**Vegetable intake**	**AOR (95% CI)**	***P* value**	**Vegetable intake**	**AOR (95% CI)**	***P* value**
AA	≤ 78 g	1.00 (Ref.)		≤ 78.4 g	1.00 (Ref.)	
	>78 g	0.86 (0.66–1.11)	0.2438	>78.4 g	1.01 (0.77–1.31)	0.9701
GA	≤ 78 g	0.70 (0.56–0.88)	0.0023	≤ 78.4 g	0.76 (0.60–0.97)	0.0237
	>78 g	0.66 (0.53–0.83)	0.0003	>78.4 g	0.73 (0.58–0.93)	0.0101
GG	≤ 78 g	0.50 (0.38–0.67)	<0.0001	≤ 78.4 g	0.72 (0.54–0.95)	0.0212
	>78 g	0.57 (0.43–0.75)	<0.0001	>78.4 g	0.65 (0.49–0.86)	0.0029

*Adjusted for age (years), region (Ansan, Ansung), smoking (none, past, current), MET (hours/week), alcohol intake (g/day), and BMI (kg/m^2^)*.

*AOR, adjusted odds ratio; 95% CI, 95% confidence intervals*.

*Vegetable intake was divided by median intake (78 g for men and 78.4 g for women)*.

### Association of Total Fruit and Vegetable Intake and rs2393791 Genotype With CRP

Associations of rs2393791 genotype and total fruit and vegetable intake divided by the median intake with elevated CRP (>0.2 mg/dL) by sex are presented in Table 6. Compared to men with the rs2393791 AA genotype who had low total fruit and vegetable intake, those with the GA or GG genotype had significantly lower inflammation levels after covariates adjustment regardless of the fruit and vegetable intake levels. The lowest Odds of having elevated CRP was shown among men with the GG genotype of rs2393791 with high fruit and vegetable intake (AOR 0.56, 95% CI 0.42–0.75). Compared to women with the rs2393791 AA genotype and low total fruit and vegetable intake, those with the GG genotype with high fruit and vegetable intake had significantly lower odds of elevated CRP (AOR 0.75, 95% CI 0.56–0.99).

**Table 6:** Association of *HNF1A* rs2393791 genotype and total fruit and vegetable intake with inflammation.

**rs2393791 genotype**	**Men (*****n*** **=** **3,700)**			**Women (*****n*** **=** **3,934)**	
	**Fruit and vegetable intake**	**AOR (95% CI)**	***P* value**	**Fruit and vegetable intake**	**AOR (95% CI)**	***P* value**
AA	≤ 224.1 g	1.00 (Ref.)		≤ 271.5. g	1.00 (Ref.)	
	>224.1 g	0.88 (0.68–1.14)	0.3180	>271.5 g	1.21 (0.92–1.58)	0.1691
GA	≤ 224.1 g	0.67 (0.53–0.84)	0.0007	≤ 271.5. g	0.74 (0.59–0.95)	0.0153
	>224.1 g	0.70 (0.56–0.88)	0.0024	>271.5 g	0.91 (0.72–1.16)	0.4476
GG	≤ 224.1 g	0.52 (0.39–0.69)	<0.0001	≤ 271.5. g	0.75 (0.56–1.00)	0.0526
	>224.1 g	0.56 (0.42–0.75)	<0.0001	>271.5 g	0.75 (0.56–0.99)	0.0466

*Adjusted for age (years), region (Ansan, Ansung), smoking (none, past, current), MET (hours/week), alcohol intake (g/day), and BMI (kg/m^2^)*.

*AOR, adjusted odds ratio; 95% CI, 95% confidence intervals*.

*Fruit and vegetable intake was divided by the median intake (224.1 g for men and 271.5 g for women)*.

## Discussion

We observed that dietary fruit and vegetable intake modified the association between the rs2393791 variants and inflammation. rs2393791 is located on chromosome 12 and intron 1 of *HNF1A*. *HNF1A* can reportedly regulate the expression of multiple target genes, including albumin secretion, anti-trypsin synthesis, and fibrinogen synthesis genes (26). Specifically, men and women with the rs2393791 GG genotype who consumed more fruit and vegetables had decreased odds of elevated CRP. When stratified by rs2393791 genotype, men harboring the G allele with high consumption of fruits and vegetables were associated with lower odds of elevated CRP. A similar finding was found in women carrying the G allele with higher vegetable intake but not with fruit intake. We also observed that an overall higher fruit and vegetable intake was associated with lower CRP levels.

We reported interactions between rs2393791 genotypes and fruit intake in relation to CRP. Higher fruit intake was inversely associated with elevated CRP, and the lowest odds of elevated CRP were found in men with rs2393791 GG genotype and high fruit intake. High fruit intake in women with the GG genotype was associated with decreased odds of elevated CRP (AOR 0.73, 95% CI 0.55–0.97), which was only slightly higher than that in those with the GG genotype and low fruit intake (AOR 0.71, 95% CI 0.53–0.95). This may be partially due to the small sample size of women who are GG carriers with elevated CRP levels and consumed fewer fruits (n = 119 out of 439; 27.1%). We observed interactions between rs2393791 genotypes and vegetable intake in relation to CRP levels. Higher vegetable intake was associated with lower CRP levels. The decrease in odds was caused by the presence of the G allele, which was stronger among those with higher vegetable intake. The lower odds of elevated CRP levels may be mediated by vegetable intake, which might be a protective factor against inflammation.

In the present study, *HNF1A* SNP rs2393791 was significantly associated with CRP levels. Specifically, the G allele was associated with lower CRP levels. The present findings indicate that five *HNF1A* SNPs (rs1920792, rs1169288, rs7310409, rs2464196, and rs1169310) are significantly associated with CRP levels in Taiwanese patients (27). CRP levels are influenced by *CRP* gene expression, which, in turn, is affected by *HNF1A* SNPs. The promoter region of the human *CRP* gene includes two different regions or acute-phase response elements. Each region includes a binding site with a low affinity for the liver-specific transcription factor HNF-1. Two HNF-1 molecules can activate *CRP* gene expression by binding simultaneously to its promoter (15). Furthermore, *HNF1A* (rs1169288) variant is associated with an increased risk of type 2 diabetes and dysglycemic status in normal-weight Japanese individuals (28). *HNF1A* genotypes for rs1169288 and rs2464196 were positively associated with total and LDL cholesterol levels in older European and American adults (29). These data suggested that *HNF1A* polymorphisms influence glucose metabolism and its related traits.

The interplay between diet and genes influences phenotypic traits. Under a hypocaloric and high-fat diet, carriers of the *HNF1A* rs7957197 T allele demonstrated a higher drop in weight loss and improved insulin resistance levels than non-carriers under the same diet (30). These findings are similar to our findings that *HNF1A* affects CRP levels by choosing different fruit and vegetable intake levels. The underlying mechanisms governing the association between *HNF1A*, dietary fruit and vegetable intake, and inflammation remain to be elucidated. However, those with a high intake of fruits and vegetables may alter the gene-association changes in inflammation.

In this cross-sectional study of Korean adults using data set from the Ansan and Ansung cohort study, “fruit” and “vegetable” dietary patterns were identified and examined in relation to CRP (31). In this study, the “vegetable” dietary pattern was only inversely associated with CRP. In contrast, no such association was observed with the “fruit” dietary pattern, indicating the difficulty in observing the isolated effect of fruits (31). Increased fruit and vegetable intake reduces inflammation (13, 32, 33). In a four-week trial, a high intake of fruits and vegetables reduced plasma CRP levels in healthy and non-smoking men. Furthermore, dietary patterns characterized by a high intake of fruits and vegetables are negatively associated with CRP levels (34–37). The inverse association between fruits and vegetables intake and CRP may be due to several nutritional components of fruits and vegetables, such as numerous phytochemicals, including flavonoids (38), which demonstrate the anti-inflammatory activity by reducing pro-inflammatory cytokines such as interleukin (IL-6), tumor necrosis factor-alpha (TNF-α), IL-1β, and cyclooxygenase-2 (COX-2), and eventually downregulating inflammatory markers such as CRP (39).

In the present study, the association between *HNF1A* genotypes and CRP differed by the intake of fruits, vegetables, and their combination. The underlying reason may be due to different amounts of flavonoids and antioxidants present in fruits and vegetables mostly consumed by Koreans in terms of lowering inflammation. Certain types of fruits, such as grapes and berries, contain high levels of antioxidants and flavonoids (40). Specifically, grapes and berry fruits have been shown to possess anti-inflammatory and antioxidative effects (40, 41). However, Koreans do not consume grapes and berry fruits as frequently. Vegetables such as garlic (42), mushroom, and white cabbage (43), which are widely consumed by Koreans, possess antioxidant properties. Fruit and vegetable items mostly consumed by Koreans contain different antioxidant properties, and these yielded the associations between CRP and *HNF1A* differ with the intake of fruits, vegetables, and their combination. Our findings indicate that men and women showed associations between CRP and *HNF1A* irrespective of fruits, vegetables, or their combination. Men and women differ substantially with respect to inflammatory regulation (44). Women demonstrate more pronounced inflammatory and innate immune responses during bacterial and viral infections than men (45). In addition, sex hormones, including estrogens, progesterone, and androgens, contribute to the differential regulation of immune responses between the sexes, and dietary factors may alter the development and functioning of the immune system differently in men and women (46).

Our study had several limitations that were considered when analyzing our findings. First, the nature of cross-sectional analyses of inflammation hinders causal inference. Second, our findings cannot be extrapolated to the entire South Korean population. The study participants resided in Ansan and Ansung in South Korea; therefore, generalizing our study findings to middle-aged Korean adults is difficult. Despite these limitations, our study had several strengths. A relatively large sample size and long follow-up period are evident in this study. Furthermore, representation of both rural and urban populations is advantageous.

In conclusion, we observed that the presence of the G allele was associated with lower CRP levels in men and women with high fruit and vegetable intake. These significant interactions may indicate that the decrease in the odds of elevated CRP levels by the rs2393791 genotype may be dependent on fruit and vegetable intake. Our findings elucidated the mechanism by which fruit and vegetable intake interact with genetic background. These novel findings indicate that the susceptibility of individuals to inflammation is affected by their dietary fruit and vegetable intake.

## Data Availability Statement

The dataset used in this study (Ansan-Ansung Cohort Study of the KoGES) was obtained after reviewing and evaluating the research plan of the Korea National Institute of Health, Korea Disease Control and Prevention Agency (http://nih.go.kr/contents.es?mid=a50401010400).

## Ethics Statement

The studies involving human participants were reviewed and approved by Institutional Review Board (IRB) of Inha University on February 18, 2022 (IRB No. 220215-1A). The patients/participants provided their written informed consent to participate in this study.

## Author Contributions

DS conducted statistical analyses and wrote the first draft of the manuscript. KL and DS conceptualized the study design, interpreted the data, and revised the manuscript. KL supervised all aspects of implementation and provided scientific advice. All authors have read and agreed to the published version of the manuscript.

## Funding

This work was supported by an Inha University Research Grant.

## Conflict of Interest

The authors declare that the research was conducted in the absence of any commercial or financial relationships that could be construed as a potential conflict of interest.

## Publisher's Note

All claims expressed in this article are solely those of the authors and do not necessarily represent those of their affiliated organizations, or those of the publisher, the editors and the reviewers. Any product that may be evaluated in this article, or claim that may be made by its manufacturer, is not guaranteed or endorsed by the publisher.
